# Is quality of care a key predictor of perinatal health care utilization and patient satisfaction in Malawi?

**DOI:** 10.1186/s12884-017-1331-7

**Published:** 2017-05-22

**Authors:** Andreea A. Creanga, Sara Gullo, Anne K. Sebert Kuhlmann, Thumbiko W. Msiska, Christine Galavotti

**Affiliations:** 1 Department of International Health, International Center for Maternal and Newborn Health, Johns Hopkins Bloomberg School of Public Health, 615 N Wolfe Street, Room E8141, Baltimore, MD 21205 USA; 2 Department of Gynecology and Obstetrics, Johns Hopkins School of Medicine, Baltimore, USA; 3 Sexual, Reproductive, and Maternal Health Team, CARE USA, Atlanta, GA USA; 4 College for Public Health and Social Justice, Saint Louis University, St. Louis, MO USA; 5 CARE Malawi, Lilongwe, Malawi

**Keywords:** Perinatal health service utilization, Quality of care, Patient satisfaction, Malawi

## Abstract

**Background:**

The Malawi government encourages early antenatal care, delivery in health facilities, and timely postnatal care. Efforts to sustain or increase current levels of perinatal service utilization may not achieve desired gains if the quality of care provided is neglected. This study examined predictors of perinatal service utilization and patients’ satisfaction with these services with a focus on quality of care.

**Methods:**

We used baseline, two-stage cluster sampling household survey data collected between November and December, 2012 before implementation of CARE’s Community Score Card© intervention in Ntcheu district, Malawi. Women with a birth during the last year (N = 1301) were asked about seeking: 1) family planning, 2) antenatal, 3) delivery, and 4) postnatal care; the quality of care received; and their overall satisfaction with the care received. Specific quality of care items were assessed for each type of service, and up to five such items per type of service were used in analyses. Separate logistic regression models were fitted to examine predictors of family planning, antenatal, delivery, and postnatal service utilization and of complete satisfaction with each of these services; all models were adjusted for women’s socio-demographic characteristics, perceptions of the closest facility to their homes, service use indicators, and quality of care items.

**Results:**

We found higher levels of perinatal service use than previously documented in Malawi (baseline antenatal care 99.4%; skilled birth attendance 97.3%; postnatal care 77.5%; current family planning use 52.8%). Almost 73% of quality of perinatal care items assessed were favorably reported by > 90% of women. Women reported high overall satisfaction (≥85%) with all types of services examined, higher for antenatal and postnatal care than for family planning and delivery care. We found significant associations between *perceived* and *actual* quality of care and both women’s use and satisfaction with the perinatal health services received.

**Conclusions:**

Quality of care is a key predictor of perinatal health service utilization and complete patient satisfaction with such services in Malawi. The current heightened attention toward perinatal health services and outcomes should be coupled with efforts to improve the *actual* quality of care offered to women in this country.

**Electronic supplementary material:**

The online version of this article (doi:10.1186/s12884-017-1331-7) contains supplementary material, which is available to authorized users.

## Background

The United Nations Millennium Development Goal 5 brought a concentrated global focus to reduce maternal mortality and ensure universal access to reproductive health [1]. Access to skilled attendants for all antenatal, delivery, and postnatal care has been heavily promoted worldwide [2], and recent surveys document an increase in use of facility-based perinatal services and considerable progress in family planning use in a majority of developing countries [1]. Yet, many now recognize that efforts to sustain or further increase use of these services in developing countries are unlikely to achieve desired gains if no attention is paid to the quality of care provided [3].

Hulton et al. have long asserted that the *actual* quality of care is only theoretical if women’s experiences receiving health services deter them from returning for subsequent care [4]. The *perceived* quality of care has been found to be an important determinant of service utilization [5, 6]. Women who perceive the quality of care in a health facility to be high are more likely to seek services there [7, 8]; conversely, perceived poor quality of care can lead to underutilization of services [9–12]. Both *actual* and *perceived* quality of care can be measured, and several frameworks for doing so have been proposed in the literature [4, 13]. Patient satisfaction is one of the most frequently reported outcome measures of quality of care [14]. It was defined as “patient’s judgment on the quality and goodness of care” [13] or “a subjective and dynamic perception of the extent to which the expected health care is received” [15], and, it is, therefore, of key interest to quality improvement in health care.

The Malawi government encourages early antenatal care, delivery in health facilities, and timely postnatal care for mothers and newborns [16]. The most recent Demographic and Health Survey conducted in Malawi found that 95% of pregnant women receive antenatal care from a skilled provider, but only 71% deliver with a skilled birth attendant and 43% receive postnatal care within the first two days after birth as recommended; also, while 65% of all women reported ever using a method of contraception, only 35% used such a method at the time of the survey [17]. A recent qualitative study assessed women’s *perceived* quality of perinatal care offered in health facilities in the country and showed that women did not know what quality of care to expect, but wanted to be well received at health facilities and treated with kindness, respect and dignity [18]. In an earlier study, Chanza et al. showed that health workers who used abusive language and had hostile behaviors deterred Malawian women from using health facilities for deliveries [19]. Similarly, another qualitative study that explored reasons why women in Malawi delivered at home without skilled attendance despite receiving antenatal care at a health centre identified health workers’ attitudes as key to women’s decision to deliver at home [20]. According to the National Reproductive Health Service Delivery guidelines in Malawi, quality of care should focus on mutual satisfaction of both patients and providers [16]. Information on Malawian women’s perceptions of the quality of perinatal care and their satisfaction with the perinatal care received in health facilities is scarce [18, 21]. Gaining a better understanding of key predictors of perinatal health service utilization and women’s satisfaction with these services can support Government efforts to ensure long-term demand for high quality perinatal care in Malawi. This study has two objectives: 1) to examine predictors of perinatal health service utilization, and 2) to assess patient satisfaction with these services when last obtained.

## Methods

### Study setting, population, and intervention

Ntcheu district is centrally located in Malawi along the border with Mozambique. Between January 2012 and December 2015, CARE implemented a Community Score Card© intervention aimed at improving utilization and quality of perinatal health services in this district [22], and used a cluster randomized design to evaluate the intervention. Both the intervention and the evaluation design are described in detail elsewhere [22]. The evaluation involved cross-sectional baseline and endline surveys of women aged 15–49 years who have given birth within the last 12 months and whose babies were alive. This analysis uses baseline data collected from 1301 women (response rate = 98%) before implementation of the intervention between November-December, 2012. The survey took 40–60 min to complete and was conducted in Chichewa in a private area of the house; all data were self-reported. Verbal informed consent was obtained from all study participants (Additional file 1).

### Analytical methods

We assessed the following population (women 15–49 years) characteristics: age, parity (1, 2, 3–4, 5+ children born), religion (Catholic, Presbyterian, other Christian, and other), Ngoni ethnicity (yes/no), marital status (married/in union or unmarried/divorced/widowed), education (completed years), reading level (cannot read simple sentence, reads part of a simple sentence, or reads the entire sentence), and household wealth (quintiles of a household wealth index constructed using principal component analysis of household item possession). Women’s perceptions of the quality of care offered in facilities closest to their residence were also examined given literature-informed expectations that: 1) if interested in obtaining perinatal health services, including family planning, women would most likely seek care at the most easily accessible facility, and 2) if the closest health facility is not perceived as providing high quality services, women will likely not seek services there and will have an overall lower likelihood of seeking services elsewhere. Specifically, we assessed perceptions regarding the cleanliness of the facility, whether the staff ensures patients’ privacy, whether one or more providers are always available at the facility, whether high quality services are offered, and whether unmarried women can access family planning and other reproductive health services there as a proxy for service accessibility. Assesment used 5-point Likert scales (strongly agree, agree, neutral, disagree, strongly disagree) and responses were dichotomized (agree or strongly agree vs. any other response) for use in analyses. In addition, we captured the time needed to reach this closest health facility using readily available means of transportation (<30 min, 30–59 min, 1–2 h, >2 h).

The perinatal health services of interest were family planning (ever and current use), antenatal care (use during the last pregnancy, pregnancy trimester of initiation during the last pregnancy, and number of visits obtained during the last pregnancy), delivery care (use at last delivery), and postnatal care (use after last delivery and number of maternal or neonatal checks within 2 months postpartum).

Survey questions to assess quality of care were developed separately for each type of health services (family planning, antenatal care, delivery care, and postnatal care) using the Hulton framework on quality of maternity care as guide (Table 3) [4]. We created binary variables for each *actual* and *perceived* quality of care items in our assessment – *yes,* if the quality aspect was reported as met; *no,* if it was not. We used the distribution of these variables to select up to five quality of care items with the highest response variability (i.e. <95% favorable responses) for each outcome; there was one exception to these criteria for the family planning outcome -- we used one item with 96.7% favorable responses because it represented a very distinct quality of care domain than the other four quality of care items chosen (i.e. whether or not the provider scheduled a follow-up visit). For family planning, of 13 quality of care items assessed, the five items chosen were: patient’s contentment with the specific method chosen (patient got the method she wanted – *yes/no*) and four elements of *actual* quality of family planning service provision used to construct an index of family planning quality provision ranging from 0 (none of the 4 quality aspects were reported) to 4 (all 4 quality aspects were reported). For antenatal care, we captured whether women received antenatal care from a skilled provider and used one of five quality of care items assessed (discussion about pregnancy danger signs). For delivery care, we recorded whether the woman had a skilled birth attendant or not, the time (in hours) before the first consult upon arrival at the facility, and used five of 12 related quality of care items to construct an index of L&D management quality; the index ranged from 0 (none of the 5 quality aspects were reported) to 5 (all 5 quality aspects were reported). For postnatal care, we captured whether women received care at the first postnatal check from a skilled provider and used one of the three quality of care items assessed for this outcome (discussion about postpartum danger signs).

Patient satisfaction with each type of service when last received was ascertained using a common question with response options measured on a 5-point Likert scale: completely unsatisfied, unsatisfied, neutral, satisfied, and completely satisfied. Given the distribution of responses, we created and used in analyses a dichotomous patient satisfaction variable (completely satisfied vs any other response) for each type of perinatal health services. Faced with the possibility of social desirability bias altering patient satisfaction responses, we attempted to validate these responses by also asking women how likely they would be to recommend the services they received to others. Responses to these questions were also measured on a 5-point Likert scale: very likely, likely, neutral, unlikely, very unlikely. We examined correlation coefficients between reported patient satisfaction and likelihood of recommending the same services to others for each type of service.

To examine predictors of perinatal health service utilization, our first study objective, we fitted logistic regression models for both ever and current use of family planning, initiation of antenatal care in 1st trimester, delivery in a health facility, and receipt of at least one postnatal check within 2 months of delivery. All models were adjusted for the socio-demographic characteristics described above with two exceptions: age and parity were highly correlated and only parity was included in all regression models; education attainment (measured in single years) and reading level were also highly correlated, and we included only reading level in all regression models. Parity was chosen over age given our interest in quality of care and women’s satisfaction with the care received -- at the population-level, we consider that parity is a better control variable for the level of maternity care that a woman needs and expects to receive. Women’s proven reading capability was preferred over education level because it is a more objective measure of a woman’s ability to obtain information on her own. All models were also adjusted for women’s perceptions of the quality of care at the closest health facility to their homes and for the time needed to reach this facility.

For the second study objective, we restricted analyses to women who have ever used family planning, used antenatal, delivery, and postnatal care during their last pregnancy, respectively. We fitted 4 logistic regression models for complete satisfaction with each of these types of services when last received. Models were adjusted for the same socio-demographic and closest health facility characteristics as well as additional factors that are theoretically expected to predict service satisfaction. Specifically, the regression model of satisfaction with family planning services was also adjusted for the ownership of the facility where services were last received (Government, private/mission-based, other), for whether the woman got the method she wanted, and the index of family planning quality provision. The model fitted for satisfaction with antenatal care services was also adjusted for the trimester when such care was initiated and the total number of visits received; for having received antenatal care from a skilled provider; and for having discussed pregnancy danger signs during antenatal care. Satisfaction with delivery care was additionally modeled on the ownership of the facility where the woman delivered (Government, private/mission-based, other), having had a skilled provider, the time to first consult before delivery, and the index of L&D management quality. The model of satisfaction with postnatal care was also adjusted for having a skilled provider, the timing of the first postnatal check given that the majority of women only had one checkup within 2 months postpartum, and having discussed postnatal danger signs with the provider.

All analyses were conducted in Stata version 13 and were adjusted for the complex survey design using Taylor’s linearization method. The research protocol was reviewed and approved by Malawi’s National Health Science Research Committee.

## Results

Of the 1301 women 15–49 years in our sample, about a quarter (26.4%) gave birth for the first time the year before the interview, while over half (52.5%) had 3 or more births (Table 1). The vast majority of women were of Ngoni ethnicity (88.7%) and married or in union (88.7%).

**Table 1:** Women’s socio-demographic characteristics and their perceptions regarding the closest health facility to their residence: Malawi, 2012

Characteristics	*N* = 1301
Women’s socio-demographic	
Age (median (range); years)	25 (15–48)
Parity (%)	
1	26.4
2	21.1
3–4	33.4
≥ 5	19.1
Religion (%)	
Catholic	23.5
Presbyterian	13.1
Other Christian	58.8
Other	4.6
Ngoni ethnicity (%)	
Yes	88.7
No	11.3
Marital status (%)	
Married/living together	88.7
Unmarried/divorced/widowed	11.3
Education (median (range); years)	4 (0–8)
Reading level (%)	
Cannot read simple sentence	29.5
Reads part of sentence	12.1
Reads the entire sentence	58.4
Household wealth (quintiles; %)	
1st (poorest)	13.1
2nd	26.1
3rd	19.9
4th	18.5
5th (richest)	22.4

Notes: All data are weighted

*FP/RH* family planning/reproductive health

Utilization of perinatal health services was high for antenatal care (99.4% attended at least one visit) and delivery care (97.3% delivered in a health facility), and relatively lower for postnatal care (77.5% had at least one postnatal check) and family planning (74.5% ever use and 52.8% current use of any contraceptive method; Table 2). However, only 16.4% of women initiated antenatal care in their first trimester, while 5.6% did so in the third trimester of pregnancy. It was at Government facilities that 84.8 and 80.1% of women last received family planning services and last delivered, respectively.

**Table 2:** Indicators of maternal and neonatal health service utilization: Malawi, 2012

Indicators		*N* = 1301
Family planning	Ever use of FP (%)	74.5
	Current use^a^ of FP (%)	52.8
	Place where FP services last obtained (%)	
	Government facility	84.8
	Private/mission-based facility	12.9
	Other	2.4
Antenatal care	Antenatal care use at last pregnancy (%)	99.4
	Trimester when ANC initiated during last pregnancy (%)	
	1st	16.4
	2nd	78.0
	3rd	5.6
	Number of ANC visits during last pregnancy (mean/std dev)	3.7 (1.1)
Delivery care	Last delivery occurred in a health facility (%)	97.3
	Facility ownership at last delivery^b^ (%)	
	Government facility	80.1
	Private/mission-based facility	19.9
Postnatal care	Postnatal care use^c^ following last delivery (%)	77.5
	Timing of 1st postnatal check^c^ (mean (std dev); weeks)	3.1 (1.7)
	Number of postnatal checks^c^ within 2 months postpartum (mean/std dev)	3.1 (0.7)

*FP* family planning, *ANC* antenatal care

Notes: All data are weighted; ^a^Among the 1281 non-pregnant, fertile women; ^b^Of those who delivered in a health facility; ^c^Maternal and/or neonatal care given interest in contact with health care system following delivery

Assessment of quality of care aspects revealed high *actual* and *perceived* quality of perinatal services. The quality of care at the closest health facility to women’s residence was generally *perceived* as high (Table 3). More than half of the women interviewed had to travel over an hour to reach the closest health facility. Nine of 13 family planning quality items, seven of 12 delivery care quality items, and all antenatal and postnatal care quality items assessed were positively reported on by more than 90% of women. Almost 90% of ever users of family planning obtained the method they wanted the last time they sought family planning services; method provision was accompanied by an average of 3.5 of the 4 items of *actual* quality of family planning provision examined (Table 4). While 96.2 and 93.0% of women obtained antenatal and postnatal care, respectively, from a skilled provider, only 91.5% of them were told about pregnancy danger signs and only 90.4% about postpartum danger signs. A high 97.5% of women who delivered in a health facility did so with a skilled birth attendant; yet, of the five L&D service quality items assessed, on average, women reported positively on 3.8 of them. Complete satisfaction with services when last received was overwhelmingly high ranging between 84.7% for delivery care and 89.3% for antenatal care. For all types of services, the distribution of women’s satisfaction with services closely matches that of women’s likelihood to recommend the same services to others (data not shown).

**Table 3:** Quality of care items used in our assessment of maternal and neonatal health services

Quality of care items assessed	%
*General perceptions on the quality of care offered at the closest health facility to the woman’s residence (N = 1301)*	
Perception that facility is clean (%)	**96.5**
Perception that staff ensures patients’ privacy (%)	**93.3**
Perception that provider(s) is(are) always available (%)	**89.8**
Perception that staff provides high quality services (%)	**89.1**
Perception that unmarried women can access FP/RH services (%)	**68.5**
Time to reach closest facility (%)	
< 30 min	**14.5**
30–59 min	**30.9**
1–2 h	**36.7**
> 2 h	**17.9**
***Family planning (N = 949)***	
** 1. Did provider mention if the method protects against HIV infection?**	**67.2**
** 2. Did provider discuss possible side effects ofthe method chosen?**	**88.3**
** 3. Did you get the method you wanted?**	**89.7**
** 4. Did provider explain how to use the method chosen?**	**94.3**
** 5. Did the provider schedule a follow-up visit?**	**96.7**
6. Did anyone at the health facility discourage you from using family planning?^a^	98.7
7. Did the health provider tell you that it was your decision whether you choose to use FP?	98.7
8. Were you given all the information or explanations you needed?	98.9
9. Were you treated with respect and dignity?	99.1
10. Did you feel the information you shared during your visit would be kept confidential?	99.3
11. Were you spoken to in a way that you could understand?	99.4
12. Were you treated with kindness and understanding?	99.5
13. Overall, did you feel it was your decision alone whether to use family planning?	99.5
***Antenatal care (N = 1291)***	
** 1. Discussion of pregnancy danger signs**	**91.5**
2. The importance of going to a health facility for antenatal checks	97.1
3. The importance of HIV testing during pregnancy	98.6
4. How to create a birth plan to prepare for the birth of your child	98.6
5. The importance of exclusive breastfeeding	98.8
***Delivery care (N = 1262)***	
** 1. Were you able to move around and choose the position that made you most comfortable?**	**51.4**
** 2. Were you involved enough in decisions about your care?**	**73.6**
** 3. Did you feel you got the pain relief you wanted?**	**78.7**
** 4. Were you left alone by midwives or doctors at a time when it worried you?** ^**a**^	**84.3**
** 5. Did the provider yell at or humiliate you in any way?** ^**a**^	**84.5**
6. Were you treated with kindness and understanding?	94.7
7. Were you spoken to in a way you could understand?	94.7
8. Were you given the information or explanations you needed?	94.8
9. Did the health provider check on you and your baby for any problems prior to discharge?	95.1
10. Were you treated with respect and dignity?	95.8
11. Did you have confidence and trust in the staff caring for you during your labor and childbirth?	96.9
12. Was the labor or delivery room you were in clean?	98.0
***Postnatal care (N = 1005)***	
** 1. Did the provider counsel you on danger signs to watch for in you and in your child?**	**90.4**
2. Did the provider give you breastfeeding support and counseling?	95.4
3. Did the provider counsel you on methods to avoid or delay another pregnancy?	97.9

Note: Bolded items were chosen for inclusion in regression analyses. ^a^Items were reverse coded; “no” responses are reported here

**Table 4:** Indicators of quality of care and satisfaction with maternal & neonatal health services when last received: Malawi, 2012

Indicators		
Family planning N = 949	Respondent got the method she wanted (%)	89.7
	Index^a^ of FP quality provision (mean/std dev)	3.5 (0.8)
	Completely satisfied with last FP services received (%)	87.6
Antenatal care N = 1291	ANC from skilled provider (%)	96.2
	Pregnancy danger signs were discussed (%)	91.5
	Completely satisfied with last ANC care received (%)	88.2
Delivery care N = 1262	Skilled birth attendant (%)	97.5
	Time to first consult before delivery mean (std dev; hours)	0.4 (2.1)
	Index^b^ of delivery service quality provision (mean/std dev)	3.8 (1.2)
	Completely satisfied with last delivery care received (%)	83.2
Postnatal care N = 1005	Skilled provider at 1st postnatal check^c^ (%)	93.0
	Postpartum^c^ danger signs were discussed (%)	90.4
	Completely satisfied with last postnatal care^c^ received (%)	87.9

*FP* family planning, *ANC* antenatal care

Notes: All data are weighted; ^a^Index constructed using 4 items: provider explained how to use chosen FP method, explained possible side effects, mentioned if method protects against HIV, and scheduled follow-up (range 0–4); ^b^Index constructed using 5 items: able to move around and choose the position that made her most comfortable, got the pain relief she wanted, not left alone by providers at a time when it worried her, provider(s) did not yell or humiliate the respondent in any way, and respondent felt involved in decision about her care (range 0–5); ^c^Maternal and/or neonatal care given interest in contact with health care system following delivery

We identified several significant predictors of perinatal health service use in our population (Table 5). Importantly, women who lived <30 min from a health facility were about half more likely (OR = 1.54; 95% CI 1.00–2.36) to have ever used family planning than those for whom the closest facility was 1–2 h away. Women who lived > 2 h than 1–2 h away from a health facility significantly decreased women’s odds of starting antenatal care in the first pregnancy trimester. Being <30 min rather than 1–2 h from a health facility more than doubled women’s odds of obtaining at least one postnatal check (OR = 2.18; 95% CI 1.34–3.55). Nulliparous women were 13 times (OR = 13.01; 95% CI 1.71–78.76) more likely than women with 3–4 births to deliver in a health facility, and compared to women in the highest wealth quintile, those in the lowest three wealth quintile had lower odds of doing so.

**Table 5:** Predictors of maternal and neonatal health service utilization: Malawi, 2012

Characteristics	Health service utilization (no = ref) -- OR (95% CI)				
	Family planning		Antenatal care initiation in 1st trimester	Delivery care	Postnatal care
	Ever use	Current use			
Socio-demographic					
Parity (3–4 = ref)					
1	**0.22 (0.15, 0.32)**	1.00 (0.74, 1.36)	1.15 (0.77, 1.70)	**13.01 (1.71, 78.76)**	1.04 (0.73, 1.47)
2	**0.61 (0.41, 0.92)**	1.07 (0.77, 1.47)	1.10 (0.73, 1.64)	0.70 (0.31, 1.54)	**1.47 (1.00, 2.18)**
≥5	1.02 (0.65, 1.61)	1.13 (0.81, 1.58)	0.69 (0.43, 1.09)	0.94 (0.39, 2.25)	1.14 (0.78, 1.65)
Religion (Other Christian = ref)					
Catholic	0.83 (0.60, 1.16)	0.94 (0.71, 1.24)	0.82 (0.56, 1.21)	0.93 (0.40, 2.19)	0.86 (0.62, 1.19)
Presbyterian	0.97 (0.63, 1.50)	*0.74 (0.52, 1.05)*	1.21 (0.79, 1.85)	0.66 (0.25, 1.76)	0.83 (0.55, 1.24)
Other	1.01 (0.54, 1.88)	*0.61 (0.34, 1.10)*	0.70 (0.31, 1.55)	0.83 (0.18, 3.80)	0.72 (0.38, 1.35)
Ngoni ethnicity (no = ref)	0.75 (0.48, 1.19)	0.85 (0.59, 1.25)	0.70 (0.45, 1.09)	0.76 (0.22, 2.62)	**0.61 (0.37, 0.99)**
Married/living together (unmarried/divorced/widowed = ref)	**3.85 (2.64, 5.61)**	**3.86 (2.58, 5.78)**	1.27 (0.76, 2.11)	1.09 (0.39, 3.09)	1.12 (0.73, 1.71)
Reading level (reads the entire sentence = ref)					
Cannot read simple sentence	**0.63 (0.46, 0.86)**	*0.78 (0.60, 1.02)*	**1.47 (1.05, 2.07)**	1.24 (0.59, 2.62)	0.93 (0.69, 1.25)
Reads part of sentence	**1.74 (1.06, 2.87)**	1.15 (0.80, 1.66)	1.10 (0.68, 1.78)	*6.94 (0.95, 50.68)*	1.13 (0.73, 1.75)
Household wealth (5th/richest = ref)					
1st (poorest)	0.89 (0.55, 1.45)	0.69 (0.45, 1.04)	0.79 (0.46, 1.37)	**0.16 (0.03, 0.80)**	0.78 (0.48, 1.25)
2nd	0.94 (0.62, 1.44)	0.77 (0.55, 1.09)	1.02 (0.65, 1.59)	**0.18 (0.04, 0.84)**	**0.64 (0.44, 0.95)**
3rd	1.04 (0.67, 1.62)	0.84 (0.59, 1.20)	1.01 (0.63, 1.61)	**0.15 (0.03, 0.72)**	0.86 (0.56, 1.32)
4th	1.02 (0.66, 1.59)	0.89 (0.62, 1.28)	1.00 (0.62, 1.60)	*0.23 (0.05, 1.19)*	0.93 (0.60, 1.44)
Closest facility to the woman’s residence					
Perception that staff provides high quality services (no = ref)	1.00 (0.60, 1.68)	1.18 (0.79, 1.77)	1.34 (0.75, 2.39)	2.04 (0.78, 5.29)	1.54 (0.98, 2.43)
Perception that staff ensures patients’ privacy (no = ref)	1.38 (0.79, 2.43)	1.35 (0.82, 2.22)	0.72 (0.39, 1.34)	0.53 (0.12, 2.42)	1.43 (0.80, 2.58)
Perception that provider(s) is always available (no = ref)	0.88 (0.52, 1.47)	1.01 (0.64, 1.58)	0.88 (0.51, 1.52)	1.65(0.52, 5.26)	0.67 (0.40, 1.12)
Perception that facility is clean (no = ref)	0.78 (0.32, 1.88)	1.00 (0.51, 1.94)	2.45 (0.84, 7.11)	n/a	1.24 (0.58, 2.65)
Perception that unmarried women can access FP services	*1.31 (0.97, 1.77)*	*1.27 (0.99, 1.62)*			
Time to reach closest facility (1–2 h = ref)					
<30 min	**1.54 (1.00, 2.36)**	1.25 (0.87, 1.80)	0.84 (0.53, 1.36)	1.39 (0.37, 5.31)	**2.18 (1.34, 3.55)**
30–59 min	1.17 (0.84, 1.64)	*1.29 (0.97, 1.71)*	0.86 (0.60, 1.23)	1.10 (0.49, 2.49)	1.10 (0.80, 1.52)
>2 h	0.96 (0.64, 1.45)	1.01 (0.73, 1.41)	**0.60 (0.38, 0.97)**	0.75 (0.30, 1.86)	0.74 (0.51, 1.08)

Notes: Multivariate logistic regression models adjusted for all factors shown and for the complex survey design; bolded figures are statistically significant at *p* < 0.05; figures shown in italics are statistically significant at *p* < 0.10; n/a, covariate predicted outcome perfectly and was dropped from model

Women with the perception that the closest facility to their home offers high quality services had significantly higher odds of being completely versus less than completely satisfied with their last antenatal care visit (OR = 1.77; 95% CI 1.00–3.12) and last delivery (OR = 1.97; 95% CI 1.20–3.26; Table 6). Women’s perception that the closest facility to their home is clean was a significant predictor of complete satisfaction with the delivery (OR = 2.85; 95% CI 1.38–5.89) and postnatal (OR = 3.53; 95% CI 1.50–8.30) care received. Relative to women who were 1–2 h away from the closest facility, those <30 min away had about half the odds of being completely satisfied with all 4 types of services; yet, those living >2 h had 2.7 times significantly higher odds of being completely satisfied with the family planning services they last received. Complete satisfaction with services appears to be strongly associated with the content of care or the *actual* quality of care received. With regard to family planning, for each one unit increase in the quality of family planning provision index, the odds of complete satisfaction with the family planning services received increased by about 50% (OR = 1.49; 95% CI 1.21–1.84). Also, for every one unit increase in the L&D management quality index, the odds of complete satisfaction with delivery services almost doubled (OR = 1.96; 95% CI 1.70–2.27).

**Table 6:** Predictors of women’s satisfaction with maternal and neonatal health services when last received: Malawi, 2012

Characteristics	Complete satisfaction with services when last received (< complete satisfaction = ref) -- OR (95% CI)			
	Family planning	Antenatal care	Delivery care	Postnatal care
Socio-demographic				
Parity (3–4 = ref)				
1	0.75 (0.41, 1.39)	0.84 (0.51, 1.39)	0.82 (0.52, 1.29)	1.05 (0.62, 1.78)
2	0.67 (0.39, 1.15)	0.87 (0.51, 1.49)	0.69 (0.43, 1.12)	0.96 (0.55, 1.66)
≥5	0.72 (0.41, 1.27)	0.64 (0.39, 1.06)	0.95 (0.57, 1.58)	0.98 (0.56, 1.74)
Religion (Other Christian = ref)				
Catholic	1.24 (0.70, 2.21)	0.95 (0.59, 1.52)	1.26 (0.82, 1.95)	1.08 (0.66, 1.77)
Presbyterian	**0.52 (0.31, 0.89)**	**0.55 (0.34, 0.91)**	0.70 (0.43, 1.14)	0.85 (0.47, 1.51)
Other	*0.43 (0.18, 1.01)*	1.06 (0.42, 2.68)	1.64 (0.69, 3.91)	1.84 (0.61, 5.62)
Ngoni ethnicity (no = ref)	*1.68 (0.94, 3.00)*	**1.78 (1.04, 3.05)**	**1.63 (1.00, 2.68)**	**1.80 (1.02, 3.16)**
Married/living together (unmarried/divorced/widowed = ref)	*1.84 (0.96, 3.51)*	1.50 (0.91, 2.48)	*0.60 (0.34, 1.08)*	1.58 (0.91, 2.74)
Reading level (reads the entire sentence = ref)				
Cannot read simple sentence	0.73 (0.45, 1.18)	0.80 (0.52, 1.24)	0.81 (0.54, 1.22)	0.91 (0.58, 1.43)
Reads part of sentence	0.85 (0.45, 1.59)	**0.59 (0.35, 0.99)**	0.66 (0.39, 1.10)	1.05 (0.56, 2.00)
Household wealth (5th/richest = ref)				
1st (poorest)	0.73 (0.39, 1.38)	0.76 (0.40, 1.44)	0.73 (0.42, 1.26)	*0.53 (0.28, 1.02)*
2nd	0.97 (0.54, 1.76)	0.66 (0.38, 1.12)	0.79 (0.47, 1.32)	*0.58 (0.32, 1.06)*
3rd	1.15 (0.60, 2.17)	0.83 (0.47, 1.47)	0.66 (0.38, 1.14)	0.82 (0.42, 1.57)
4th	1.22 (0.62, 2.38)	1.21 (0.65, 2.25)	0.73 (0.43, 1.24)	0.89 (0.46, 1.74)
Closest facility to the woman’s residence				
Perception that staff provides high quality services (no = ref)	1.06 (0.52, 2.15)	**1.77 (1.00, 3.12)**	**1.97 (1.20, 3.26)**	1.54 (0.75, 3.14)
Perception that staff ensures patients’ privacy (no = ref)	0.94 (0.39, 2.24)	0.56 (0.25, 1.30)	0.78 (0.42, 1.46)	**0.24 (0.07, 0.78)**
Perception that provider(s) is always available (no = ref)	1.33 (0.67, 2.68)	*1.65 (0.95, 2.87)*	1.31 (0.79, 2.16)	0.99 (0.51, 1.92)
Perception that facility is clean (no = ref)	*2.21 (0.91, 5.35)*	1.91 (0.80, 4.57)	**2.85 (1.38, 5.89)**	**3.53 (1.50, 8.30)**
Perception that unmarried women can access FP services	*0.65 (0.41, 1.05)*			
Time to reach closest facility (1–2 h = ref)				
<30 min	**0.53 (0.31, 0.93)**	**0.46 (0.27, 0.80)**	**0.54 (0.32, 0.89)**	**0.53 (0.31, 0.91)**
30–59 min	0.83 (0.51, 1.36)	*0.65 (0.41, 1.04)*	1.02 (0.66, 1.58)	1.10 (0.65, 1.87)
>2 h	**2.71 (1.18, 6.21)**	0.66 (0.38, 1.13)	1.13 (0.69, 1.84)	0.90 (0.49, 1.66)
Maternal health service utilization indicators				
Place where FP services last obtained (Government facility = ref)^a^ Private/mission-based facility	1.57 (0.77, 3.23)			
Trimester when ANC initiated last pregnancy (2nd = ref)				
1st		1.05 (0.64, 1.73)		
3rd		0.63 (0.30, 1.12)		
Number of ANC visits last pregnancy		0.94 (0.78, 1.12)		
Facility ownership last delivery (Government facility = ref) Private/mission-based facility			1.36 (.086, 2.15)	
Timing of 1st postnatal check^b^ (weeks)				1.01 (0.89, 1.15)
Number of checks within 2 months postpartum^b^				1.04 (0.77, 1.42)
Quality of services when last received				
Respondent got the FP method she wanted (no = ref)	*0.80 (0.63, 1.03)*			
Index^c^ of FP quality provision	**1.49 (1.21, 1.84)**			
ANC from skilled provider (no = ref)		0.44 (0.14, 1.38)		
Pregnancy danger signs were discussed (no = ref)		1.29 (0.70, 2.36)		
Skilled birth attendant (no = ref)			0.51 (0.20, 1.33)	
Time to first consult before delivery (hours)			1.03 (0.95, 1.12)	
Index^d^ of delivery service quality provision			**1.96 (1.70, 2.27)**	
Skilled provider at 1st postnatal check^b^ (no = ref)				0.40 (0.14, 1.17)
Postpartum^b^ danger signs were discussed (no = ref)				**2.65 (1.46, 4.80)**

*FP* family planning, *ANC* antenatal care

Notes: Results from multivariate logistic regression models adjusted for all factors shown and for the complex survey design; figures in bold are statistically significant at *p* < 0.05; figures shown in italics are statistically significant at *p* < 0.10; ^a^Also controlled for other place where family planning services were obtained; ^b^Maternal and/or neonatal care given interest in contact with health care system following delivery; ^c^Index constructed using 4 items: provider explained how to use chosen FP method, explained possible side effects, mentioned if method protects against HIV, and scheduled follow-up (range 0–4); ^d^Index constructed using 5 items: able to move around and choose the position that made her most comfortable, got the pain relief she wanted, not left alone by providers at a time when it worried her, provider(s) did not yell or humiliate the respondent in any way, and respondent felt involved in decision about her care (range 0–5)

## Discussion

We found higher levels of perinatal health service use in Ntcheu district at the end of 2012 than documented by the 2010 Malawi Demographic and Health Survey, yet in line with the 2014 Malawi MDG Endline Survey [23]. Thus, there is now need to not only further increase use of perinatal services, including family planning, but also to sustain current levels of use in Ntcheu district to meet women’s needs. We found that married women were about 4 times more likely to be ever or current users of family planning, while women of lower parity were less likely to be ever or current users of family planning. Targeting unmarried and low parity women with family planning messages and counseling may lead to an increase in contraceptive use prevalence and healthy spacing of births in these subgroups of women.

Proximity to a health facility was significantly associated with women’s use of perinatal services. Women who lived <30 min compared to those 1–2 h from a health facility had significantly higher odds of being ever users of family planning and of using postnatal care; conversely, those residing >2 h compared to 1–2 h from a health facility were less likely to initiate antenatal care in their first trimester of pregnancy. These findings highlight the need for lower level health facilities, not only district and referral hospitals, to be capable to offer counseling, basic emergency obstetric care, family planning, and neonatal health services as well as referrals to district hospitals when complications occur.

The importance of early initiation of antenatal care should be stressed at every medical encounter with women of reproductive age. In addition, we found that nulliparous women were significantly more likely to deliver with a skilled birth attendant when compared to their counterparts. This may be the beginning of a generational change in delivery practices in Malawi whereby increasingly higher percentages of nulliparous women deliver in a health facility with a skilled attendant and, depending on their experiences, may continue this practice at a 2nd and higher order birth. However, when compared to women in the richest wealth quintile, those in the three poorest wealth quintiles were significantly less likely to have a skilled birth attendant. Clearly, without addressing inequities in perinatal care access, making progress in increasing the use of perinatal services may not be a realistic goal despite government policies requiring that women deliver in health facilities and banning traditional birth attendants from assisting with deliveries.

Women reported high overall satisfaction (≥85%) with all perinatal services examined, higher for antenatal and postnatal care than for family planning and delivery care, thus in line with results from other studies that found women to report overall greater satisfaction with their antenatal than delivery care [24]. The equally high reported likelihood of recommending the perinatal services received to others provides some reassurance with regard to the validity of women’s reports of service satisfaction. Other studies in developing countries, while using other measures, found similarly high satisfaction with perinatal services based on women’s self-reports. For example, Changole et al. found that most (97.3%) women who delivered at Queen Elizabeth Central Hospital in Blantyre, the largest hospital in Malawi, reported being satisfied with the care offered [21]. A study conducted in Bangladesh indicated that the level of maternal satisfaction on delivery care was 92.3% [25]. In Ethiopia, the overall satisfaction with delivery services in a recent small town survey was 81.7% [26], slightly higher than corresponding figures reported by an older study conducted in Amhara Referral Hospitals (61.9%) and Assela Hospital (80.7%) [27]. However, only 51.9 and 56.0% of women interviewed for two studies conducted in South Africa and Kenya, respectively, were satisfied with their delivery services [28, 29].

Satisfaction with care does not necessarily equate to receipt of good quality of care – women may not be aware of the standard of care and may have low or no expectations. As others also note, it is unusual for a woman to feel completely satisfied with every aspect of her care during pregnancy, labor and delivery, and postpartum [30]. Many would report being completely satisfied with the quality of care received, but can easily identify aspects of care that they disliked [31]. Our assessment of specific items of perinatal care quality revealed interesting findings. Notably, 24 of 33 (73%) quality of perinatal care items assessed were positively reported on by more than 90% of women. These findings should, however, be interpreted with caution in light of results from studies that compared women’s reports of care with direct observation of care. For example, Rosen et al. used structured, standardized clinical observation checklists to directly observe quality of care at facilities in five African countries -- while women in all five countries reported being treated with dignity and in a supportive manner by providers, some actually experienced poor interactions with them [3]. Thus, reports of *perceived* quality of care may not accurately reflect the *actual* quality of care.

Nevertheless, reports of *actual* quality of care items can be used to improve provider training programs in the district. For family planning, nine in 10 women received the contraceptive method of choice, were counseled on correct method use and possible side-effects, and had a follow-up visit scheduled during the office visit; yet, only two thirds of women were counseled on whether or not their method of choice offered protection against HIV infection the last time they sought family planning services. These results indicate a higher quality of family planning provision in Ntcheu district than documented by the 2013 Malawi Service Provision Assessment at the national level [32] –the latter found that most family planning consultations included discussions of client concerns about her contraceptive method (75%), but fewer included discussions about side effects (44%) and only 23% of consultations had any discussion related to STIs. With regard to delivery care, only about half of women reported being able to move around and deliver in their preferred position, about 3 in 4 felt involved in their care, about 4 in 5 got the pain relief they wanted, and 85% were not yelled at or humiliated and were not left alone at a time when it worried them. These findings closely match results from other studies [3, 33]. For example, the most frequently mentioned form of disrespect in the study by Rosen et al. was abandonment and neglect during labor, and the same study reported lack of adequate pain relief for some participants [3].

The World Health Organization recommends monitoring and evaluation of maternal satisfaction to improve the quality and efficiency of health care during childbirth [14]. Our analysis identified significant predictors of women’s satisfaction with the perinatal health services received in Ntcheu district, Malawi. Key among these were quality of care elements. A *perceived* high quality of care being offered at the closest health facility increased the odds of women reporting complete vs less than complete satisfaction with the antenatal and delivery services received. Also, the perception that the closest health facility was clean increased the odds of women reporting complete vs less than complete satisfaction with both delivery and postnatal services. While we did not record where these services were actually obtained, with more than half of women having to travel more than an hour to the closest health facility, this was likely the place where care had been obtained. We also found strong associations between elements of *actual* quality of care and women’s satisfaction with the care received. This serves as validation for the measures we used to examine actual quality of perinatal care (i.e. family planning quality index, several items in the L&D management quality index, danger sign discussions during antenatal and postnatal care), and also as indication that women can likely recognize and value high quality services. Notably, the higher the family planning quality index and the L&D management quality index, the significantly more likely women were to report complete vs less than complete satisfaction with family planning and delivery services received, respectively; also, having discussed postnatal danger signs with a provider significantly increased the likelihood of reporting complete vs less than complete satisfaction with postnatal care. On the other hand, receipt of services from a skilled provider did not increase women’s odds of reporting complete satisfaction with services. This may be due to women’s expectation to have skilled providers available in health facilities, which has already become the norm. Somewhat surprisingly, relative to women who were 1–2 h away from the closest health facility, those <30 min away had about half the odds of being completely satisfied with all four types of perinatal health services; yet, those living >2 h had 2.7 times significantly higher odds of being completely satisfied with the family planning services they last received. It may be that the closest facilities were lower level and/or lower quality facilities and women who traveled longer appreciated getting the services they want; with regard to family planning, those who travel longer value the opportunity of getting their preferred method.

The study is not without limitations. It is representative of catchment areas of 20 of the 33 health facilities in Ntcheu district [22], but may not be representative of the entire district or other districts in Malawi. All data are self-reported by the women interviewed. Information about the timing when each type of service was last received was not recorded, thus there is risk of recall bias. Also, responses are prone to social desirability bias, which is possible given the high rates of reported service utilization and satisfaction with services. We did not rely on validated measures of quality of care perceptions, yet used culturally sensitive questions to yield relevant and easy to measure quality of care indicators. Of note, our survey questions did not separately capture information on maternal versus neonatal checks within 2 months postpartum; thus, we cannot draw conclusions for use, predictors of use, and patient satisfaction with postnatal services separately for mothers and infants.

## Conclusions

Our study has important implications. In Ntcheu district, there is a need to promote initiation of antenatal care early in pregnancy not only to improve the likelihood of women being well informed and for pregnancy complications, if they occur, to be identified early, but also in light of evidence that high quality information received from providers during pregnancy results in favorable pregnancy outcomes [18]. Health workers in all types of facilities have a responsibility to inform women about the care they should expect, while the quality of perinatal care and women’s satisfaction with such should continue to be examined in Malawi. With over 80% of women delivering and receiving family planning services in public facilities, the Ministry of Health in Malawi should strengthen these facilities and ensure that good quality perinatal care is available for all women irrespective of socio-economic status. The current heightened attention paid to maternal and neonatal health service utilization as well as to maternal and neonatal morbidity and mortality outcomes should be coupled with a keen interest in understanding women’s satisfaction with the services received antepartum, intrapartum, and postpartum – all are key to improving quality of care for pregnant, delivering, and postpartum women in Malawi.

## Additional file

Additional file 1: Data file. (XLSX 372 kb)

## Abbreviations

ANC: Antenatal care
FP: Family planning
FP/RH: Family planning/reproductive health
GVH: Group villages
HIV: Human immunodeficiency virus
L&D: Labor and delivery
STIs: Sexually transmitted infections
